# Ultrasonographic misdiagnosis of multicystic mesothelioma of the omentum: A case report

**DOI:** 10.1097/MD.0000000000030441

**Published:** 2022-09-09

**Authors:** Yuhong Diao, Li Chen, Zhixing Liu

**Affiliations:** a Department of Ultrasonography, First Affiliated Hospital of Nanchang University, Nanchang, China.

**Keywords:** greater omentum, multicystic mesothelioma, ultrasonic diagnosis

## Abstract

Multicystic Mesothelioma (MM) is very rare and preoperative diagnosis is difficult.

**Patient concerns:**

We report a case of a 34-year-old man with solid abdominal cystic echo mass. Physical examination showed that the patient had a flat and soft abdomen without tenderness or rebound pain, no fluid wave tremor, and no obvious abdominal mass was touched. The patient complained of repeated abdominal distention with nausea for 5 days. Sonographic examination suspected pseudomyxoma peritoneum.

**Diagnosis:**

Conventional ultrasound examination showed a cystic solid echo mass in the right abdominal cavity of the patient, with uneven internal echo and honeycomb change, and clear boundary with surrounding organs. Color Doppler suggested that the blood flow in the mass was not obvious. Contrast-enhanced computed tomography of the abdomen revealed hypodensity foci in hepatic and renal crypts and right paracolic sulcus.

**Interventions:**

Laparoscopic resection of the mass was performed, and the postoperative pathological findings were polycystic mesothelioma (greater omentum).

**Outcomes:**

After mass resection, all laboratory tests and abdominal ultrasound were normal, and abdominal distension and nausea disappeared.

**Lessons:**

Improved ultrasound diagnosis of MM is useful for clinical decision-making.

## 1. Introduction

Multicystic mesothelioma (MM) is a rare benign tumor of Mesothelioma originating from mesenchymal cells. It occurs in the pleura, pericardium, or peritoneal space and is most common in young and middle-aged women.^[1,2]^ Since 1979, it has been found by Mennemeyer et al.^[3]^ So far, only more than 200 cases have been reported worldwide, most of which are individual cases.^[4]^

Polycystic mesothelioma is rare and preoperative diagnosis is mostly based on imaging examination, but none of them is specific. Herein, we report a case of polycystic mesothelioma of the omentum that was correctly diagnosed after laparoscopic mass resection.

## 2. Case presentation

**Figure 1.**
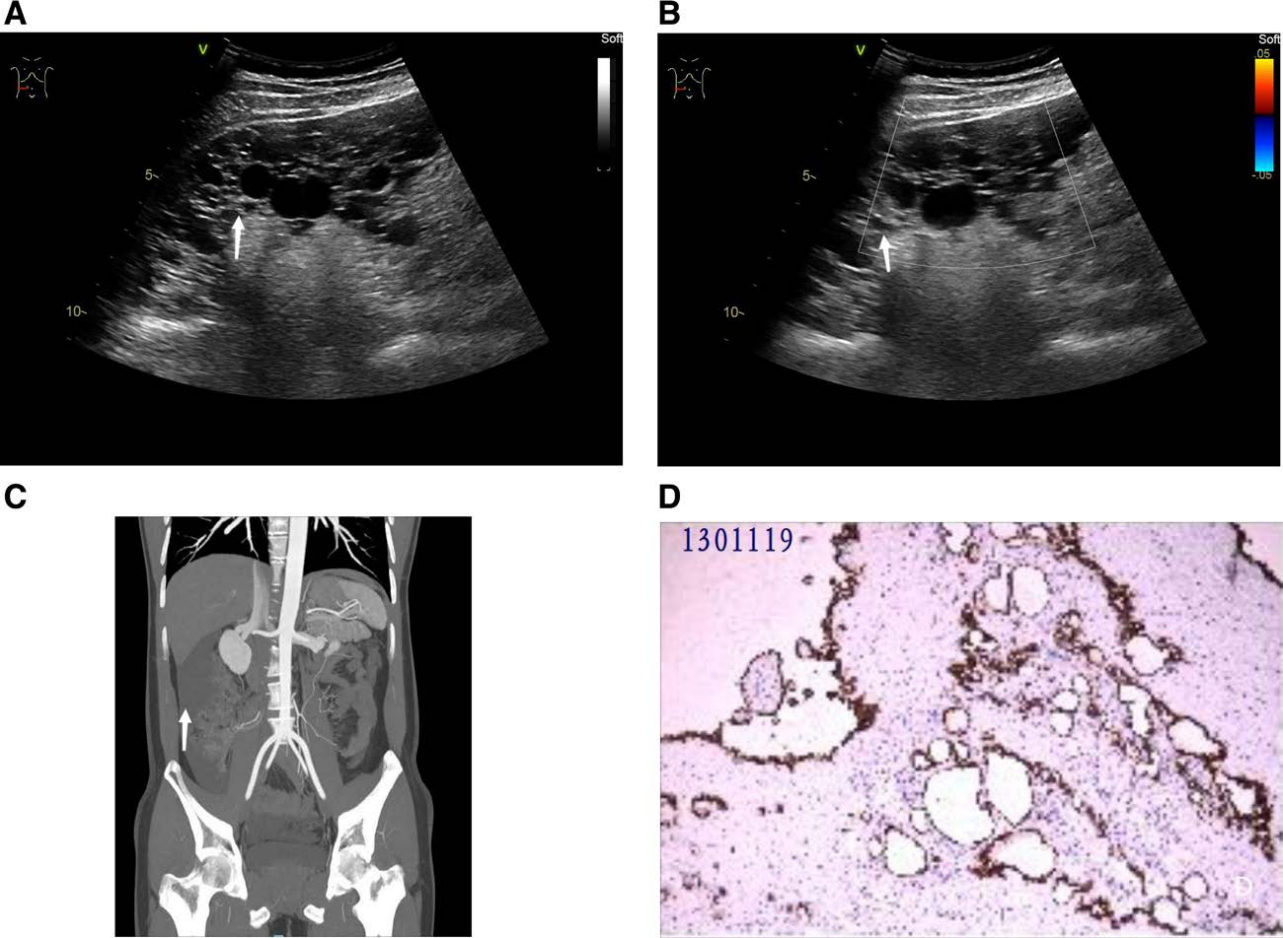
A 34-year-old male patient with Multicystic mesothelioma of the omentum. (A) Ultrasound of an abdominal mass (arrow); (B) No obvious blood flow signal (arrow) Color Doppler ultrasound image in abdominal mass; (C) CT of an abdominal mass (arrow); (D) Pathological map of an abdominal mass (HE, ×200). CT = contrast-enhanced computed tomography.

A 34-year-old man presented to our hospital with recurrent abdominal distention and nausea for 5 days. Physical examination found that the patient’s abdomen was flat and soft, without tenderness or rebound pain, without fluid wave tremor, and no obvious abdominal mass was touched. Conventional ultrasound examination found that cystic solid echo mass could be seen in the abdominal cavity of the patient on the right side, ranging from the liver and kidney space to the horizontal line of the ischial spine. The internal echo was uneven and showed cheese sign change, and the boundary of liver, kidney, and colon was clear. Color Doppler ultrasound suggested that the blood flow in the abdominal mass was not obvious (Fig. 1A and B). Ultrasound suggested solid abdominal sac mass, and the possibility of pseudomyxoma peritoneum was considered. Contrast-enhanced computed tomography of the abdomen revealed low-density foci in hepatic and renal crypts and right paracolic sulcus, the nature of which was undetermined, and the possibility of pseudomyxoma peritoneum was not excluded (Fig. 1C). For surgical treatment, preoperative tumor markers CEA, CA125, CA199, and AFP were negative. Laparoscopic mass resection was performed. During the operation, there were strong jelly-like tissues in the abdominal cavity, which were beaded and had an unclear boundary with omentum tissue, and were widely distributed. The gross appearance of the tumor was about 12 × 12 × 3.8 cm grayish yellow broken tissue with several vesicles about 0.1 to 1.5 cm in diameter, containing clear fluid. The postoperative pathological diagnosis was polycystic mesothelioma (greater omentum). Microscopically, multiple luminal structures of varying sizes were seen in the fibrous tissue (Fig. 1D). Six months after the operation, the patient had no recurrence and showed no symptoms.

## 3. Discussion

MM is a rare benign tumor derived from Mesothelioma cells, with an estimated incidence of 2 in 1000,000 per year.^[5]^ The prognosis after surgical resection is excellent, but the recurrence rate is high and malignant transformation has been reported in a few cases.^[6–8]^ MM usually occurs in the pleura, pericardium, or peritoneal space, and is more common on the surface of pelvic organs in young and middle-aged women. Since 1979, it has been found by Mennemeyer et al,^[3]^ only more than 200 cases have been reported in the world,^[4]^ and most of them are individual cases. At present, the pathogenesis of MM is still unclear, and the nature of the tumor and reactivity have been controversial.^[6,9,10]^ Its pathogenesis may include: chronic stimulation, such as pelvic inflammatory disease, endometriosis, and previous abdominal surgery, leads to reactive proliferation of mesenchymal cells^[6,7,11–15]^; the disease occurs mostly in young and middle-aged women, so it is speculated that sex hormones are caused by the disease^[11]^; genetic factors.^[16–19]^

Histologically, the mesenchymal cells lining cysts are varied in shape.^[20]^ Cysts are filled with serous fluid, and the stroma between cysts may contain inflammatory cells and fibrous components.^[6]^ The diagnosis is usually large in diameter and maybe multifocal, free-floating, or unilocular or multilocular with surrounding structures. It usually occurs in women, but also men and children.^[9,21]^ Its clinical symptoms have no obvious specificity,^[22]^ and most of them are discomforts such as abdominal distension, abdominal pain, and palpable abdominal mass caused by the compression of the intestinal tract or other abdominal organs due to the increase of tumor volume.^[23–25]^ Dyspareunia, constipation, dysuria, and/or frequent urination are rare, weight loss or familial Mediterranean fever is rare,^[20]^ and ascites may occur when cyst masses get close and stick together. Complete resection of cystic lesions is the first line of treatment and can avoid local recurrence.^[20]^

Preoperative diagnosis were mostly based on imaging examination, but none of them had specificity. Compared with contrast-enhanced computed tomography, ultrasound has many advantages, such as better fine resolution and dynamic observation of the relationship between tumor and surrounding tissue, so it is the preferred method for the diagnosis of this disease. The ultrasonic characteristics of MM are large cystic or cystic solid masses, with thin and smooth walls and no echo in the fluid. The banded separated or dense spot-like echo can be seen in some parts. Peritoneal cystic mesothelioma around the ovary exhibits a “spider web” sign.^[22]^

The ultrasound features of this patient were different from those reported in the literature. In this case, several small anechoic areas were observed in the tumor with cheese sign changes and good internal acoustic permeability, indicating that the cystic fluid composition was single, possibly some leaking fluid, which was different from mucus composition.

The incidence of MM is low and clinical understanding is insufficient, so it should be differentiated from intraperitoneal cystic lymphangioma, pseudomyxoma, cystic adenoma of the ovary, and other cystic masses during ultrasound diagnosis. Cystic lymphangioma is often located in the retroperitoneum with an irregular shape and grows along with the tissue space under the extrusion of the retroperitoneal organs. The ultrasonic manifestation of pseudomyxoma in the abdominal cavity was heterogeneous hypoechoic ascites. The ascites were mostly gelatinous and surrounded by the abdominal organs, and blood flow signals were visible in the ascites. Ovarian cystadenoma occurs in the pelvic adjunct area. Papillary substantive echo could be seen in the serous cystadenoma wall, while mucinous cystadenoma has a unilateral multi-compartment structure with poor internal acoustic permeability, which is helpful to distinguish it from abdominal MM.

Strengthening the preoperative recognition of this rare disease is beneficial for patients to make individualized methods to achieve clinical treatment.^[26]^ Therefore, an ultrasound found intraperitoneal polycystic mass with the cheese sign change, thin capsule wall, and good acoustic transmission in the echo-free area, which should be considered for the diagnosis of intraperitoneal polycystic mesothelioma. However, histological evaluation is still necessary when MM cannot be differentiated from other cystic lesions.

## Author contributions

**Conceptualization:** Zhixing Liu.

Data curation: Yuhong Diao.

Investigation: Li Chen.

Writing – original draft: Yuhong Diao.

Writing – review & editing: Zhixing Liu.
